# Prevalence of chronic kidney diseases and its determinants among Iranian adults: results of the first phase of Shahedieh cohort study

**DOI:** 10.1186/s12882-022-02832-5

**Published:** 2022-06-09

**Authors:** Ali Dehghani, Sadegh Alishavandi, Nader Nourimajalan, Hossein Fallahzadeh, Vahid Rahmanian

**Affiliations:** 1 Department of Epidemiology and Biostatistics, School of Public Health, Shahid Sadoughi University of Medical Sciences and Health Services, Yazd, Iran; 2 Division of Nephrology, Department of Internal Medicine, Shahid Sadoughi University of Medical Sciences, Yazd, Iran; 3 Research Center of Prevention and Epidemiology of Non-Communicable Disease, Department of Biostatistics and Epidemiology, Shahid Sadoughi University of Medical Sciences, Yazd, Iran; 4 Research Center for Social Determinants of Health, Jahrom University of Medical Sciences, Jahrom, IR Iran

**Keywords:** Chronic kidney disease, Attributable risk, Hypertriglyceridemia, Hypercholesterolemia

## Abstract

**Background:**

Chronic kidney disease (CKD) is one of the major global causes of mortality, described as the most neglected chronic disease. This study aimed to determine the prevalence and determinants of CKD in the setting of the Shahedieh cohort study in Yazd, Iran.

**Methods:**

This cross-sectional study was conducted on adults in the baseline phase of the Shahedieh cohort study in Yazd, Iran. In this study, 9781 participants aged 30–73-year-old were investigated. The data used in this study included demographic and clinical variables and blood samples. Adjusted odds ratios were employed using multivariate logistic regression; meanwhile, population attributable risks for CKD were calculated and reported.

**Results:**

CKD prevalence was 27.5% (95%CI: 26.57–28.34) in all participants, 24% in male, and 30.3% in female. The results of multivariate logistic regression analysis identified age (OR = 1.89, 95%CI:1.082–1.96), women (OR = 1.62, 95%CI: 1.45–1.79), BMI ≥ 30 (OR = 1.40,95%CI: 1.20–1.62), diabetes (OR = 1.38, 95%CI: 1.22–1.57), hypertriglyceridemia(OR = 1.20, 95%CI: 1.01–1.43), history of cardiovascular disease (OR = 1.20, 95%CI: 1.01–1.43), hypertension (OR = 1.18, 95%CI: 1.04–1.33), smoking (OR = 1.17, 95% CI: 1.02–1.33), LDL ≥ 130 (OR = 1.15, 95%CI: 1.01–1.31), history of kidney stone (OR = 1.14, 95%CI: 1.01–1.32) and hypercholesterolemia (OR = 1.14, 95%CI: 1.01–1.32) as risk factors for CKD. Among individual factors, obesity (11.25%), Hypertriglyceridemia (9.21%), LDL ≥ 130 (7.12%) had the greatest Population-Attributable Fraction, followed by Hypercholesterolemia (5.2%), diabetes (5.05%), smoking (3.73%) and high blood pressure (2.82%).

**Conclusion:**

The results showed that the main determinants of CKD are potentially modifiable risk factors. Therefore, implementing early detection and screening programs in people at risk as well as preventive measures such as lifestyle modification programs and risk factors controlling can prevent the disease.

## Introduction

Chronic kidney disease (CKD) is one of the major causes of mortality worldwide [1, 2]. The number of deaths due to CKD has increased by 82.3% in the past two decades, about the third-largest increase among 25 leading causes of mortality, only second to HIV/AIDS (396%) and diabetes [1, 3]. CKD, as the 27th mortality cause in 1990, rose to the 18th mortality cause in 2010 [3]. CKD claimed the lives of about 1 million in 2013 [4] and 1.2 million in 2017 [5].

Despite global concerns, CKD disproportionately impacts the developing countries [6], affecting 14.3 and 13.4% of low- and middle-income countries and the global population, respectively [2]. The WHO projections indicate that the CKD mortality will steadily increase to 14 deaths in 100,000 general population in 2030 [7].

Currently, the number of patients with an end-stage renal disease requiring kidney replacement therapy is estimated globally at 1.4 million with an annual growth rate of 8% [8]. With an aging population, the sharp rise in the prevalence of type 2 diabetes and hypertension augments this growth rate, placing a colossal burden on health resources [1]. Despite the discovery of new renal biomarkers for a better clinical diagnosis and understanding of the pathophysiology of different diseases, about 13.3 million patients develop renal insufficiency annually, of whom 85% live in developing countries [9].

CKD is defined as an abnormality of kidney structure or function, present for > 3 months, with health implications and CKD is classified based on cause, glomerular filtration rate (GFR), and albuminuria categories (CGA). CKD progression defined based on one or more of the following items: Decline in GFR category: (≥ 90[G1: Normal or high], 60–89 [G2: Mildly decreased], 45–59 [G3a: Mildly to moderately decreased], 30–44 [G3b: Moderately to severely decreased] 15–29[G4: Severely decreased], < 15 [G5: Kidney failure] ml/min/ 1.73 m^2^) [10].

CKD burden is more common in low- and middle-income countries, accounting for about 80% of the total CKD cases. In a meta-analysis study (2016) on the global prevalence of chronic kidney disease, this was estimated at 11.6% in Iran (4.51 to 18.84%) [11]. According to a study by Moazzeni et al., 2021, in Iran, about 8.4 and 9.3% of women and men, respectively, develop CKD annually [12].

Some studies have identified risk factors for CKD entailing age, sex, ethnicity, family history of CKD, socioeconomic status, metabolic syndrome, urinary albumin excretion, dyslipidemia, nephrotoxins (NSAIDs, antibiotics, radiological contrast), primary kidney disease, urinary disorders (obstruction, recurrent urinary tract infections), cardiovascular disease, diabetes mellitus and acute kidney disorders (AKD) [13–15].

Considering the high prevalence of CKD and its financial burden, more up-to-date studies are required to determine the disease status, which will be of benefit in terms of strategic planning for the prevention and management of CKD [7]. This study aimed to determine the prevalence and determinants of CKD in the setting of the Shahedieh cohort study in Yazd, Iran.

## Materials and methods

### Study design and population

The present cross-sectional study was conducted using phase one of an observational prospective cohort study of the 30–73 years-old population of Shahediyeh, Yazd, Iran. This was a part of the PERSIAN, Prospective Epidemiological Research Studies in Iran, multicenter cohort study conducted in 2016 aiming at the evaluation of non-communicable diseases such as diabetes and its risk factors. This region was selected due to various factors such as accessibility, lack of migration to other cities, partially homogeneous ethnicity of the residents, and the cooperation of local people. Comprehensive information regarding the protocol of the PERSIAN cohort study is provided elsewhere [16]. The study was started on 05 May 2015, and then the enrollment phase was concluded in Sep 2016. In summary, participants were selected by multi-stage cluster random sampling after they provided informed consent. Informed consent for illiterate participants, was obtained from their legally authorized representatives. In the PERSIAN cohort study, blood samples were taken from eligible participants. Demographic characteristics and lifestyle-related information such as diet and smoking were assessed through valid questionnaires. Meanwhile, anthropometric indices and blood pressure were measured for all participants. All information was collected via professional interviewers [16].

### Data collection

**Fig. 1:**
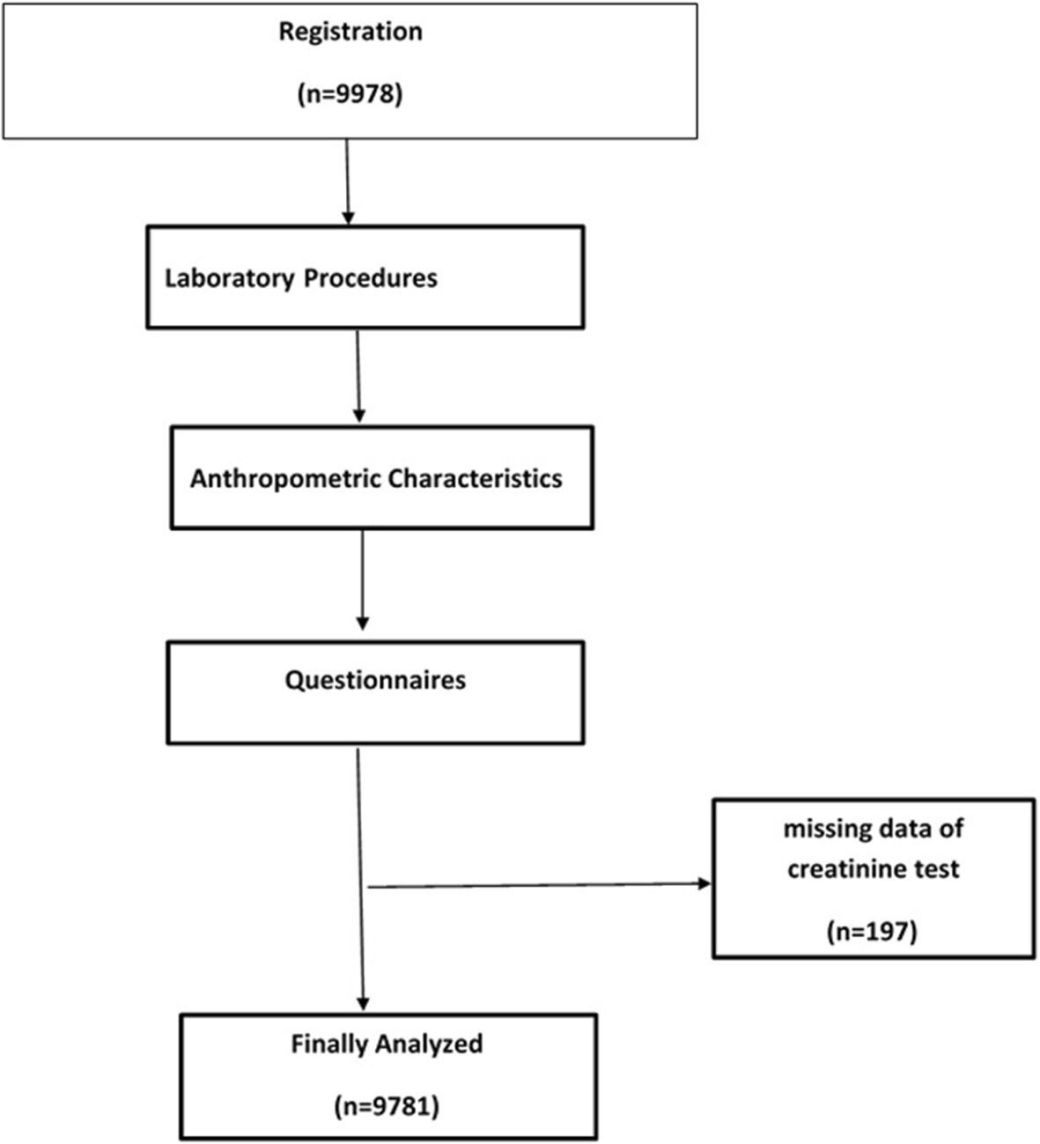
Study flowchart

The data of 9978 subjects were collected using questionnaires, clinical examination, urine and blood tests, and para-clinical tests. The characteristics of the participants including age, sex, education level, marital status, occupation, anthropometric indices (BMI, waist to hip ratio), amount of water consumption, history of renal stones, dyslipidemia, smoking, hypertension, diabetes, and self-reported history of cardiovascular diseases (heart disease and/or stroke) were extracted from Shahediyeh Cohort Study. A creatinine test results were included in the study, and those without creatinine test results were excluded leading to the remaining 9781 subjects with creatinine test results (Fig. 1).

### Measures

Normal weight was specified as BMI less than 25 kg/m^2^, overweight by 25 to < 30 kg/m^2^ and obese as ≥30 kg/m^2^ [17]. In this study, high blood pressure is defined as an SBP ≥140 mmHg or DBP ≥ 90 mmHg, and/or the consumption of antihypertensive medications. Diabetes was defined as self-reported diabetes or intake of blood glucose-lowering medications or having an FBS ≥126 mg/dL. Hypercholesterolemia and hypertriglyceridemia were considered as serum total cholesterol (TC) and triglyceride of 240 and 200 mg/dl or more or receiving lipid-lowering drugs as The US National Cholesterol Education Programme Adult Treatment Panel III (NCEP ATP III), respectively [18]. Furthermore, an LDL level of ≥130 mg/dl was defined as high LDL.

Serum creatinine levels were measured according to the standard colorimetric Jaffe-Kinetic reaction method [19]. The estimated glomerular filtration rate (eGFR) was calculated by the subsequent equation [10]:

$$141\ \min {\left(\mathrm{SCr}/\mathrm{k},1\right)}^{\mathrm{a}}\ \max \left(\mathrm{SCr}/\mathrm{k},1\right){-}^{1.209}\ {0.993}^{\mathrm{Age}}\ \left[\ 1.018\ \mathrm{if}\ \mathrm{female}\right]\ \left[\ 1.159\ \mathrm{if}\ \mathrm{black}\right],\mathrm{where}\ \mathrm{SCr}\ \mathrm{is}\ \mathrm{serum}\ \mathrm{creatinine}\ \left(\mathrm{in}\ \mathrm{mg}/\mathrm{dl}\right),\mathrm{k}\ \mathrm{is}\ 0.7\ \mathrm{for}\ \mathrm{female}\mathrm{s}\ \mathrm{and}\ 0.9\ \mathrm{for}\ \mathrm{males},\kern0.5em \mathrm{a}\ \mathrm{is}-0.329\ \mathrm{for}\ \mathrm{female}\mathrm{s}\ \mathrm{and}-0.411\ \mathrm{for}\ \mathrm{males},\min\ \mathrm{is}\ \mathrm{the}\ \mathrm{minimum}\ \mathrm{of}\ \mathrm{SCr}/\mathrm{k}\ \mathrm{or}\ 1,\mathrm{and}\ \max\ \mathrm{is}\ \mathrm{the}\ \mathrm{maximum}\ \mathrm{of}\ \mathrm{SCr}/\mathrm{k}\ \mathrm{or}\ 1.5$$

We defined CKD in this study by one measurement of serum creatinine and eGFR less than 60 ml/ min/1.73m^2^ we used Standard National Health Interview Survey (NHIS) current cigarette smoking variable definition [20]. The first question, asked of all participants, is “have you smoked at least 100 cigarettes in your entire life?” participants answering “yes” are classified as ever smokers, and those who answer “no” are classified as never smokers. Ever smokers are then asked a second question: “do you now smoke cigarettes every day, some days or not at all?” participants who answer “every day” or “some days” are classified as current smoker. Likewise, people with a history of opium and alcohol use were considered to be exposed to these risk factors [21, 22].

### Statistical analysis

Data were analyzed using SPSS software (version 20.0). Qualitative data are presented as numbers and percentages; meanwhile, quantitative data are reported as mean and standard deviation. Chi-square was applied to evaluate the association or difference between qualitative variables.

Univariate logistic regression analyses were performed to identify risk factors of CKD. In the subsequent stage, variables with *p* ≤ 0.2 were enrolled in the multivariate logistic backward elimination technique to identify the involved variables. In this technique, first by entering the variables in the equation and then based on the fact that the independent variable that is removed from the equation does not cause a significant increase in the value of R squared (R2), that variable is removed from the equation. In the same way, the remaining independent variables are tested until removing a variable from the equation causes a significant reduction in R2, in which case the analysis is completed.

The results were expressed by odds ratios with a 95% confidence interval. A *p*-value less than 0.05 was considered statistically significant. The population attributable risk was estimated using the following formula [23]:

$$\%{AR}_{pop}=\frac{Pe\left( OR-1\right)}{1+ Pe\left( OR-1\right)}\times 100$$

Where OR is the odds ratio of the risk factor calculated using multivariate logistic regression analysis and, P_e_ is the ratio of the population exposed to the risk factor. 95% confidence intervals were computed using standard error estimates for attributable risk [24] using SAS Version 9.1 (SAS Institute, Cary, NC).

## Results

In total, 9781 subjects aged 30–73 years were evaluated in this study, with a mean age of 48.39 ± 9.58 years, and the sex of participants was equally distributed (*p* = 0.65). In terms of education, 31.3% had primary education, 20.1% had diploma and separately 16.6% had illiteracy and intermediate education, respectively. 95.7% of the participants were married and 4424 (42.33%) had a job. The mean BMI and W/H ratio were 28.4 ± 4.87 and 0.93 ± 0.07, respectively. The prevalence of smoking, opium use, and alcohol consumption were 22.1, 10.9 and 3.8%, respectively. Moreover, the prevalence of hypertension, diabetes, history of renal stone, and history of CVD and the history of stroke was 20.8, 17.5, 15.9, 7.8, 2.1 and 1.1%, respectively (Table 1).

**Table 1:** Frequency of study variables based on CKD Status on adult Shahedieh residents aged 30–73 years

Variable	Category	Total (*N* = 9781)	CKD Status		P-value
			CKD (*N* = 2685)	Non-CKD (*N* = 7096)	
Age (yr), mean (SD)	–	48.39(9.58)	54.09(9.42)	46.30(8.073)	< 0.0001*
waist-to-hip ratio, mean (SD)	–	0.93(0.07)	0.94(0.07)	0.092(0.06)	< 0.0001*
Sex, frequency (percent)	Male	4921(50.3)	1186(44.2)	3735(52.6)	< 0.0001^**^
	Female	4860(49.7)	1499(55.8)	3361(47.4)	
Level of Education, frequency (percent)	Illiterate	1619(16.6)	744(27.7)	875(12.3)	< 0.0001^**^
	Primary	3063(31.3)	904(33.6)	2159(30.4)	
	Intermediate	1620(16.6)	344(12.8)	1276 (18)	
	Diploma	1968(20.1)	384(14.3)	1584(22.3)	
	Associate Degree	378(3.9)	95(3.5)	283(4)	
	Bachelor	936(9.6)	174(6.5)	762(10.7)	
	Master’s and higher	197(2.0)	40(1.4)	157(2.2)	
BMI, frequency (percent)	< 25	2355 (24)	513(19)	1798(25.3)	< 0.0001^**^
	25–29.9	4086(42)	1148 (43)	3052(43)	
	≥30	3340(34)	1024 (38)	2246(31.7)	
marital status, frequency (percent)	Not married	37(0.4)	8(.3)	26(.4)	< 0.0001^**^
	Married	9358(95.7)	2507(93.3)	6861(96.7)	
	Widow	339(3.4)	155(5.8)	180(2.5)	
	Divorced	47(0.5)	15(.6)	29(.4)	
Smoking, frequency (percent)	Yes	2210(22.6)	540(20.1)	1670(23.6)	< 0.0001^**^
	NO	7571(77.4)	2145(79.9)	5426(76.4)	
Employed, frequency (percent)	Yes	4424(42.3)	857(32)	3567(50.3)	< 0.0001^**^
	NO	5357(57.7)	1828(68)	3529(49.7)	
Diabetes, frequency (percent)	Yes	1781(17.5)	735(27.4)	1046 (14)	< 0.0001^**^
	NO	8000(82.5)	1950(70.7)	6050(85.2)	
High blood pressure, frequency (percent)	Yes	2071(20.8)	898(33.5)	1173(16.5)	< 0.0001^**^
	NO	7710(79.2)	1787(66.5)	5923(83.5)	
history of CVD, frequency (percent)	Yes	783(7.8)	349(13)	434(6.1)	< 0.001^**^
	NO	8998(92.2)	2336(87)	6662(93.9)	
History of myocardial infarction, frequency (percent)	Yes	213(2.1)	88(3.3)	125(1.8)	< 0.0001^**^
	NO	9568(97.9)	2597(96.7)	6971(98.2)	
History of stroke, frequency (percent)	Yes	110(1.1)	56(2.1)	54(.8)	< 0.0001^**^
	NO	9671(98.9)	2629(97.9)	7042(99.2)	
History of renal stones, frequency (percent)	Yes	1583(16.2)	518(19.3)	1065 (15)	< 0.0001^**^
	NO	8198(83.8)	2167(80.7)	6031(85)	
Opium consumption, frequency (percent)	Yes	1040(10.6)	233(8.7)	807(11.4)	< 0.0001^**^
	NO	8741(89.4)	2452(91.3)	6289(88.6)	
Alcohol consumption, frequency (percent)	Yes	366(3.8)	93(3.5)	273(3.8)	0.353^**^
	NO	9415(96.2)	2592(96.5)	6823(96.2.)	
TG, frequency (percent)	≥200	4556(46.6)	1424 (53)	3132(44.1)	< 0.0001^**^
	< 200	5225(53.4)	1261 (47)	3964(55.9)	
LDL, frequency (percent)	≥130	5156(52.7)	1528(56.9)	3628(51.1)	< 0.0001^**^
	< 130	4625(47.3)	1157(43.1)	3468(48.9)	
HDL, frequency (percent)	< 35	574(5.9)	136(5.1)	438(6.2)	< 0.0001^**^
	≥35	9207(94.1)	2549(94.9)	6658(93.8)	
Chol, frequency (percent)	≥240	3602(36.9)	1167(43.5)	2435(34.3)	< 0.0001^**^
	< 240	6179(63.1)	1518(56.5)	4661(65.7)	
Water consumption per day, frequency (percent)	8 glass >	9503(97.1)	2617(97.5)	6886(97)	0.523^**^
	> 8 glass	278(2.9)	68(2.5)	210(3)	

*LDL* low-density lipoproteins, *HDL* high-density lipoprotein, *TG* Triglyceride, Cholesterol (Chol), *BMI* Body mass index, *CVD* cardiovascular diseases, *SD* Standard Deviation

^*^independent t-test

^**^chi-squared test, significance level < 0.05

Mean serum cholesterol, LDL, HDL and TG were 189.72 ± 40.47, 103.90 ± 32.68, 52.71 ± 12.21 and 166.36 ± 10.1.57 mg/dl, respectively. The mean of blood urea nitrogen (BUN) and creatinine (CR) were 28.08 ± 8.49 and 1.13 ± 0.26 mg/dl, respectively. The mean of the Glomerular filtration rate (GFR) was 68.66 ± 14.54.

Besides, the prevalence of CKD was 27.5% (95%CI: 26.57–28.34), in all participants (*n* = 2685), 24% in male, and 30.3% in female. The mean age of participants in the CKD group was higher than in the non-CKD group (*p* < 0.0001) (Table 1).

The result of the univariate analysis showed that age, sex, education, BMI, waist to hip ratio, smoking, diabetes, high blood pressure, opium use, employment status TG, LDL, HDL, cholesterol and the history of myocardial infarction, stroke, renal stones and CVD were associated with CKD in the participants (*P* < 0.05) (Table 2).

**Table 2:** The risk factors could predict CKD by uni-variate logistic regression

Variable	Category	B Coefficient	Crude Odds Ratio (OR) (95%CI)	***P***-value
Age (yr)	–	0.089	1.09(1.08–1.11)	< 0.0001
waist-to-hip ratio	–	1.06	2.89(2.004–4.39)	< 0.0001
Sex, Male/ Female	–	0.34	1.40(1.28–1.53)	< 0.0001
Level of Education	Illiterate	–	Ref	–
	Primary	−0.70	0.49(0.43–0.55)	< 0.0001
	Intermediate	−1.14	0.31(0.27–0.37)	< 0.0001
	Diploma	−1.25	0.28(0.24–0.33)	< 0.0001
	Associate Degree	−0.92	0.39(0.30–0.50)	< 0.0001
	Bachelor	−1.31	0.26(0.22–0.32)	< 0.0001
	Master’s and higher	−1.23	0.29(.20–0.42)	< 0.0001
BMI	< 25	–	Ref	–
	25–29.9	0.27	1.32(1.17–1.48)	< 0.0001
	≥30	0.46	1.59(1.41–1.80)	< 0.0001
marital status	Not married	–	Ref	–
	Married	−0.519	0.595(.217–1.63	0.313
	Widow	−0.348	0.706(.378–1.32)	0.276
	Divorced	0.510	1.665(.861–3.219)	0.13
Smoking Yes/No	–	0.169	1.184(1.061–1.322)	.003
Employed No/Yes	–	0.778	2.177(1.980–2.392)	< 0.0001
Diabetes Yes/No	–	0.589	1.802(1.615–2.011)	< 0.0001
High blood pressure Yes/No	–	0.967	2.629(2.373–2.913)	< 0.0001
History of CVD Yes/No	–	0.856	2.353(2.026–2.733)	< 0.0001
History of myocardial infarction Yes/No	–	0.667	1.948(1.475–2.573)	< 0.0001
History of stroke Yes/No	–	1.076	2.934(2.002–4.299)	< 0.0001
History of renal stones Yes/No	–	0.326	1.386(1.233–1.557)	< 0.0001
Opium consumption Yes/No	–	−0.306	0.736(.632–.858)	< 0.0001
Alcohol consumption Yes/No	–	−0.114	0.893(.702–1.134)	0.353
TG	≥200	0.357	1.429(1.307–1.562)	< 0.0001
	< 200	–	Ref	–
LDL	≥130	0.354	1.42(1.27–1.58)	< 0.0001
	< 130	–	Ref	–
HDL	< 35		1.23(1.012–1.50)	0.038
	≥35	–	Ref	–
Chol	> 240	0.386	1.42(1.34–1.611)	< 0.0001
	< 240	–	Ref	–
Water consumption per day	8 glass >	0.159	1.17(0.88–1.54)	0.260
	> 8 glass	–	Ref	–

*OR* Odds Ratio, “- “Not applicable, *LDL* low-density lipoproteins, *HDL* high-density lipoprotein, *TG* Triglyceride, *Chol* Cholesterol, *BMI* Body mass index, *CVD* cardiovascular diseases, *Ref* Reference group, significance level < 0.05

In the next step, all variables but marital status, alcohol and water consumption were subjected to variable selection in multivariate logistic regression analysis.

According to the results of multivariate analysis, an obese participant has 1.4 times higher odds of having CKD than a person with a BMI < 25 (OR = 1.40,95%CI:1.20–1.62), a diabetic person has 1.38 times higher odds of having CKD than a person without diabetes (OR = 1.38,95%CI:1.22–1.57), a person with triglyceride > 200 has 1.23 times higher odds of having CKD than a person triglyceride < 200 (OR = 1.23,95%CI:1.10–1.26), a person with history of CVD has 1.20 times higher odds of having CKD than a person without history of CVD (OR = 1.20,95%CI:1.01–1.43), a person with hypertension has 1.18 times higher odds of having CKD than a person without hypertension (OR = 1.18,95%CI:1.04–1.33), a person smoker has1.17 times higher odds of having CKD than a person never smokers (OR = 1.17,95% CI: 1.02–1.33), a person with LDL ≥ 130 has 1.15 times higher odds of having CKD than with LDL < 130 (OR = 1.15,95%CI:1.01–1.31), a person with history of kidney stone has 1.14 times higher odds of having CKD than a person without history of kidney stone (OR = 1.14,95%CI:1.01–1.32) and a person with cholesterol level ≥ 240 has1.14 times higher odds of having CKD than with cholesterol level < 240 (OR = 1.14,95%CI:1.01–1.32) (Table 3).

**Table 3:** The risk factors could predict CKD by multivariate logistic regression analysis

Variable	Category	B Coefficient	Adjusted OR (95%CI)	*P*-value
Age (yr)	–	0.085	1.89(1.082–1.096)	< 0.0001
waist-to-hip ratio	–	0.175	1.20(1.01–1.53)	0.046
Smoking, Yes/No	–	0.157	1.17(1.02–1.33)	0.021
Employed, No/Yes	–	0.101	1.10(1.04–1.17)	0.001
Sex, Male/ Female	–	.482	1.62 (1.45–1.79)	< 0.0001
Diabetes, Yes/No	–	0.326	1.38(1.22–1.57)	< 0.0001
High blood pressure Yes/No	–	0 .168	1.18(1.04–1.33)	0.008
History of CVD Yes/No	–	.188	1.2(1.01–1.43)	.036
History of renal stones Yes/No	–	0.182	1.14(1.07–1.30)	0.039
Hypertriglyceridemia	≥200	0.209	1.23(1.10–1.26)	< 0.0001
	< 200	–	Ref	–
Hypercholesterolemia	≥240	0.150	1.16(1.01–1.32)	.028
	< 240	–	Ref	–
Low Density Lipoprotein (LDL)	≥130	.144	1.15(1.011–1.319)	.034
	< 130	–	Ref	–
BMI	< 25	–	Ref	–
	25–29.9	0.200	1.22(1.06–1.40)	0.005
	≥30	0.388	1.40(1.20–1.62)	< 0.0001

*OR* Odds Ratio, *BMI* Body mass index, *CVD* cardiovascular diseases, “- “Not applicable, *Ref* Reference group, significance level < 0.05

**Fig. 2:**
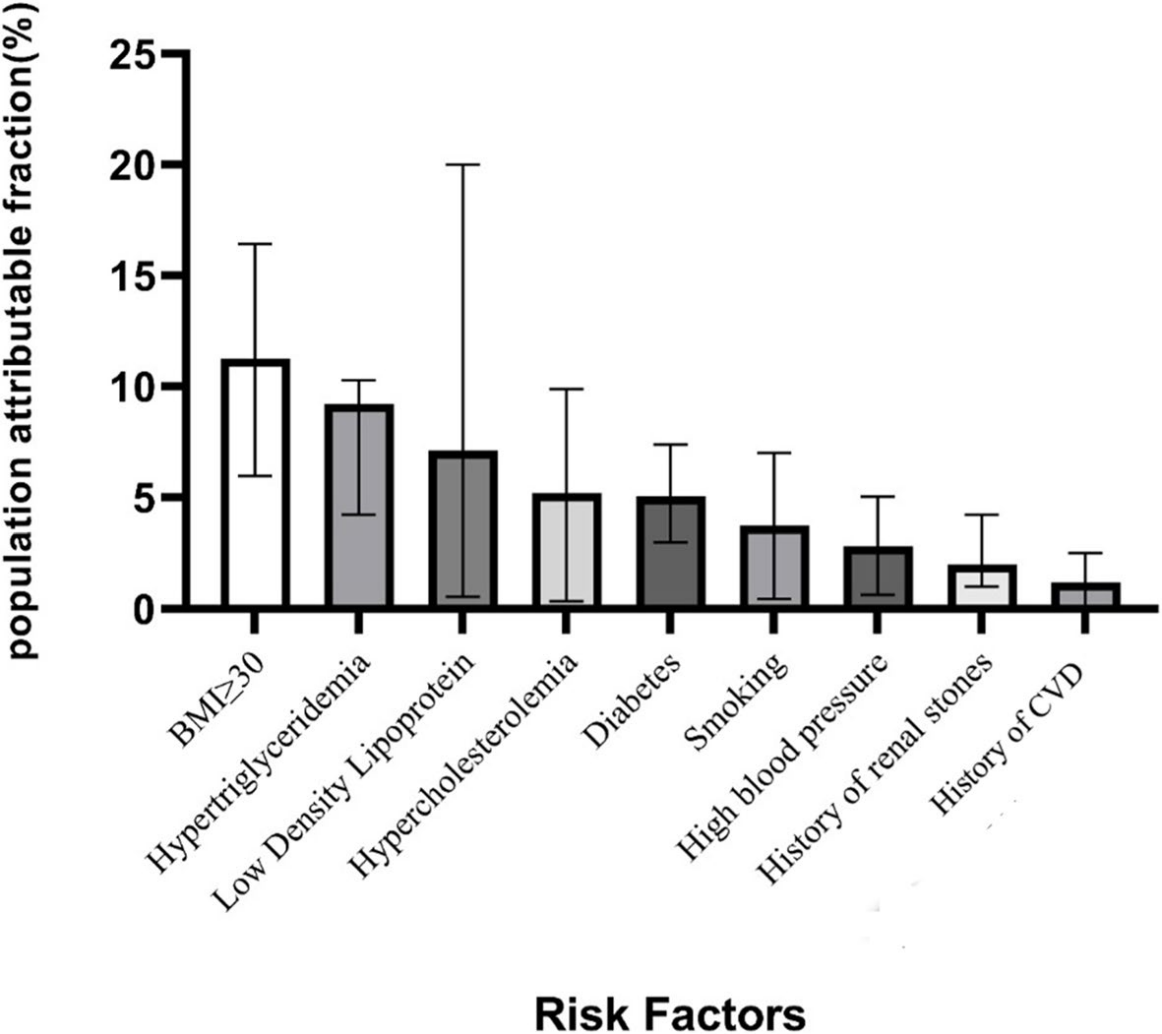
Population attributable fraction and 95% confidence intervals of CKD due to modifiable risk factors

The population attributable risk for modifiable risk factors of CKD were BMI ≥ 30 (PAF11.25, 95%CI: 5.96–16.43), Hypertriglyceridemia (PAF 9.21, 95%CI: 4.22–10.29), Low Density Lipoprotein (PAF7.12, 95%CI: 0.56–14.02), Hypercholesterolemia (PAF 5.2, 95%CI: 0.34–9.89), Diabetes (PAF5.05, 95%CI: 2.99–7.39), Smoking (PAF 3.73, 95%CI:0.45–7), High blood pressure (PAF 2.82, 95%CI: 0.64–5.04), History of renal stones (PAF 2.02, 95%CI: 1.02–4.22) and the history of CVD (PAF 1.19, 95%CI: 0.06–2.52) (Fig. 2).

## Discussion

The results of this study showed that CKD prevalence was 27.5% in the 30–73-year-old population of Yazd, 24% in men and 30.3% in women. Female sex, older age, BMI, diabetes, hypertriglyceridemia, hypertension, smoking, LDL ≥ 130, hypercholesterolemia and the history of kidney stone, as well as CVD, were all pertinent to CKD.

In a cross-sectional study, Safarinejad et al. reported that CKD prevalence was 12.6% among 17,000 adults ≥14 years old, selected from 2002 to 2005 [25]. Najafi et al. reported that CKD prevalence among adults 18 years and older in Golestan based on GFR was 4.6% In 2008 [26], in their subsequent report, based on GFR and albuminuria, it increased to 8.89% [27]. Naqibi et al. reported a prevalence of 5.1% in Gonbad in 2012 [28]. According to eGFR, CKD prevalence was reported about 16.6% in a study in Shiraz, Iran (2019) [29]. In the Golestan cohort study (as the largest cohort in Iran and the Middle East) by Sepandloo et al. (2017), CKD prevalence was estimated at 23.7% (26.6% in women and 20.6% in men) [19]. Our estimates indicated a higher prevalence compared to previous reports. However, no significant conclusions can be drawn regarding CKD prevalence in Iran, because previous studies in variable environments have been performed on participants in the variable age range. Our estimates are relatively higher than similar reports among adults in American and European countries [30, 31]. Of note, the mean age of participants in this study was 48 years, and apparently, the mean eGFR in this group would be lower than in younger age ones. In addition, the relatively high prevalence of CKD in Yazd could be due to the high prevalence of hypertension (20.8%), diabetes (17.5%), overweight (41.9%) and obesity (34.5%) in this area.

Glomerular filtration rate (GFR) has been shown to decline with age after maturity [32]. The multivariate analysis results demonstrated that CKD chance elevates by 1.89 with 1 year aging, which was consistent with previous studies indicating an increase in the odds of CKD with age [2, 19, 33]. Poggio et al, in the study of 1057 healthy subjects, showed, GFR declined by 4 mL/min/1.73 m^2^ per decade up to the age of 45 years, and 8 mL/min/1.73 m^2^ per decade thereafter [34]. Blake et al in one study showed the decline in GFR with age was nonlinear, getting steeper with age [35].

Other result of this study showed that a male participant has 1.62 times higher odds of having CKD than female. Most of the studies have reported higher odds of CKD in women [2, 19, 33, 36, 37]. One study (2019) reported that the male sex was associated with a reduced eGFR mean [38]. In addition, sex differences can be related to sex hormones and specific sex genetics [39].

We found a strong association between BMI, waist-to-hip ratio and CKD. This finding was similar to the results of the Golestan cohort study (as the largest cohort in Iran and the Middle East) conducted in 2017 [19]. Furthermore, Wang et al. showed that overweighting and obesity without metabolic abnormality are accompanied by a higher risk of incident CKD compared with those with normal body weight and devoid of metabolic abnormality [40]. These findings may have imperative implications for clinical performance and public health, and it has been suggested that preventing weight gain is as pivotal as reducing current weight [41].

In the present study, CKD had a significant association with diabetes, and a diabetic person has 1.38 times higher odds of having CKD than a person without diabetes. Diabetic patients are at risk for renal injuries due to reduced renal reserve [42]. Compared to previous studies on this subject, there are several plausible explanations for the elevation of CKD incidence among the diabetic population of Iran. First, about 50 and 30% of the Iranian diabetic population had achieved treatment goals for hyperglycemia and hypertension, respectively [43]. In fact, many patients with DM were poorly monitored, which may help the increase of diabetes complications, such as CKD. Second, unhealthy diets [44], more particularly, high salt intake [45], are prevalent among the Iranian population and are speculated as a risk factor for CKD [46]. Third, it has been reported that urbanization factors are associated with chronic kidney disease [45]. Since, the study population is limited to Yazd city, the higher CKD incidence in our study can be explained to some extent.

Hypertension is common in patients with CKD [47]. The prevalence ranges from 60 to 90%, depending on the stage of CKD and its cause [48]. This study’s results showed that hypertension prevalence in CKD patients was 33.4%, and a person with hypertension (having a blood pressure of BP ≥140/90 mmHg or taking antihypertensive drugs) has 1.18 times higher odds of having CKD than a person without hypertension. In some previous cohort studies on diabetic populations, hypertension was an independent predictor of CKD [2, 19, 29–31, 49]. The mechanisms of hypertension in CKD include volume overload, sympathetic overactivity, salt retention, endothelial dysfunction, and alterations in hormonal systems that regulate blood pressure (BP) [47]. Kokubo et al. have shown that high blood pressure increases the incidence of CVD in people with CKD more than in patients with normal kidney function [50]. CKD, in turn, even in its early stages, can eventuate in high blood pressure and an elevated risk of CVD. The association between CKD and CVD has been extensively demonstrated in previous studies [51–53]. CKD shares risk factors with CVD such as smoking, overweight status, high blood pressure, diabetes, hyperglycemia and elevated cholesterol. Further studies are demanded to the profound perception of risk factors for CVD in cases with CKD, which is likely to be accompanied by the development of preventive and therapeutic strategies to diminish life-threatening complications and high mortality in individuals affected by CKD [54, 55].

The effect of a kidney stone on its dysfunction has not been widely considered in epidemiological studies. CKD is more prevalent in patients with kidney stones. About 10 to 15% of these patients will eventually develop chronic kidney failure [56]. This study’s results showed that having a history of kidney stones is likely to elevate the risk of chronic kidney disease by 14%. A cohort study (2015) [57] and a meta-analysis study (2017) [58] showed that a positive history of kidney stones increased the risk of CKD by 29 and 47%, respectively.

The present study showed that the odds of having CKD were 1.23 and 1.14 times higher in participants with hypertriglyceridemia and hypercholesterolemia than healthy individuals, respectively. One study found that the association between hypertriglyceridemia and CKD [59]. In another study, a significant correlation was found between high LDL and cholesterol levels and CKD [60].

In the present study, we did not find any association between the amount of daily water intake and CKD. Optimal daily water intake to impede chronic kidney disease (CKD) progression is unknown. In a study in 2021, Wagner et al. showed that the relation between plain water intake and progression to kidney failure might be U-shaped. Low and high intake may not have merits for CKD [61]. Clark et al. in a pilot randomized controlled trial reported that there were no significant changes in urine, serum osmolality or electrolyte concentrations, or eGFR between two groups of receiving adjusted water intake and control groups [62]. However, some studies found a significant association between water intake and CKD [2, 63], in which high water intake was considered as a protective factor against CKD compared to low water intake [2, 64].

The present study found no association between CKD and education level or marital status, which was in accordance with previous studies [2, 29]. Even so, the Golestan Cohort Study found an indirect association between CKD and education level [2, 19].

### Strengths and limitations

This was the study to be conducted on CKD prevalence and its associated risk factors on a large sample size of Iranian adults using standardized measurement procedures as well as an extensive range of potential risk factors.

Apart from the above-mentioned strengths, there were some limitations in our study. First, although the study sample is based on population, but apparently does not represent the whole country and includes most of the Fars community in Iran in specific time. Second, it is impossible to show a causal relationship by virtue of the fact that this was a cross-sectional study. Therefore, more prospective studies are demanded in this area. Third, chronic kidney disease was defined based on a creatinine test, and we did not repeat the test 3 months later. Fourth, due to the lack of serum uric acid results and information on socioeconomic factors, these two variables were not studied.

## Conclusion

The results showed that the main risk factors of CKD are potentially modifiable ones. It is possible to deter CKD occurrence to some extent through lowering the lipid levels, controlling blood pressure and blood sugar in diabetic and hypertensive patients. Since CKD prevalence elevates with age, screening for CKD among individuals more than 50 years is suggested to be integrated into the primary healthcare system. This strategy, in turn, leads to diagnosing the disease in early stages to prevent progression to kidney failure and kidney replacement therapy. Therefore, it necessitates making decisions to promote the knowledge and awareness of health staff as well as the general population regarding early diagnosis and the prevention of CKD to improve its prognosis and impede mortality and unnecessary financial burdens associated with kidney failure.

## Data Availability

Data for this study were obtained from the Shahediyeh cohort study, Yazd, Iran, which is part of the PERSIAN multicenter cohort study conducted in 2016 in Iran. All data used in this study were obtained from the Shahid Sadoughi University of Medical Silences website (http://web.ssu.ac.ir/page-shahedia2/fa/24/form/pId16856). The research data used to support the findings of this study are available from the corresponding author of this study upon request.

## Abbreviations

CKD: Chronic kidney disease
LDL: Low-density lipoproteins
HDL: High-density lipoprotein
TG: Triglyceride
Chol: Cholesterol
BMI: Body mass index
GFR: Glomerular Filtration Rate
Cr: Creatinine
CVD: Cardiovascular Disease
NHIS: National Health Interview Survey
NCEP ATP III: The US National Cholesterol Education Programme Adult Treatment Panel III

## References

[1] Radhakrishnan J, Remuzzi G, Saran R, Williams DE, Rios-Burrows N, Powe N (2014). Taming the chronic kidney disease epidemic: a global view of surveillance efforts. Kidney Int.

[2] Ene-Iordache B, Perico N, Bikbov B, Carminati S, Remuzzi A, Perna A (2016). Chronic kidney disease and cardiovascular risk in six regions of the world (ISN-KDDC): a cross-sectional study. Lancet Glob Health.

[3] Lozano R, Naghavi M, Foreman K, Lim S, Shibuya K, Aboyans V (2012). Global and regional mortality from 235 causes of death for 20 age groups in 1990 and 2010: a systematic analysis for the global burden of disease study 2010. Lancet.

[4] Snively CS, Gutierrez C (2004). Chronic kidney disease: prevention and treatment of common complications. Am Fam Physician.

[5] Bikbov B, Purcell CA, Levey AS, Smith M, Abdoli A, Abebe M (2020). Global, regional, and national burden of chronic kidney disease, 1990–2017: a systematic analysis for the global burden of disease study 2017. Lancet.

[6] Mills KT, Xu Y, Zhang W, Bundy JD, Chen C-S, Kelly TN (2015). A systematic analysis of worldwide population-based data on the global burden of chronic kidney disease in 2010. Kidney Int.

[7] Webster AC, Nagler EV, Morton RL, Masson P (2017). Chronic kidney disease. Lancet.

[8] White SL, Chadban SJ, Jan S, Chapman JR, Cass A (2008). How can we achieve global equity in provision of renal replacement therapy?. Bull World Health Organ.

[9] Raimann JG, Riella MC, Levin NW (2018). International Society of Nephrology’s 0by25 initiative (zero preventable deaths from acute kidney injury by 2025): focus on diagnosis of acute kidney injury in low-income countries. Clin Kidney J.

[10] Outcomes KDIG, Group CW (2013). KDIGO 2012 clinical practice guideline for the evaluation and management of chronic kidney disease. Kidney Int.

[11] Hill NR, Fatoba ST, Oke JL, Hirst JA, O’Callaghan CA, Lasserson DS (2016). Global prevalence of chronic kidney disease–a systematic review and meta-analysis. PLoS One.

[12] Moazzeni SS, Arani RH, Hasheminia M, Tohidi M, Azizi F, Hadaegh F (2021). High incidence of chronic kidney disease among Iranian diabetic adults: using CKD-EPI and MDRD equations for estimated glomerular filtration rate. Diabetes Metab J..

[13] Noble R, Taal MW (2019). Epidemiology and causes of chronic kidney disease. Medicine..

[14] Taal M, Brenner B (2006). Predicting initiation and progression of chronic kidney disease: developing renal risk scores. Kidney Int.

[15] Kalantar-Zadeh K, Jafar TH, Nitsch D, Neuen BL, Perkovic V (2021). Chronic kidney disease. Lancet.

[16] Poustchi H, Eghtesad S, Kamangar F, Etemadi A, Keshtkar A-A, Hekmatdoost A (2018). Prospective epidemiological research studies in Iran (the PERSIAN cohort study): rationale, objectives, and design. Am J Epidemiol.

[17] Ren H, Zhang L, Liu Z, Zhou X, Yuan G (2019). Sleep duration and apolipoprotein B in metabolically healthy and unhealthy overweight/obese phenotypes: a cross-sectional study in Chinese adults. BMJ Open.

[18] National Cholesterol Education Program (US). Expert panel on detection, evaluation, and treatment of high blood cholesterol in adults. Executive summary of the third report of the National Cholesterol Education Program (NCEP) expert panel on detection, evaluation, and treatment of high blood cholesterol in adults (adult treatment panel III). JAMA. 2001;285:2486–97. 10.1001/jama.285.19.2486 11368702

[19] Sepanlou SG, Barahimi H, Najafi I, Kamangar F, Poustchi H, Shakeri R (2017). Prevalence and determinants of chronic kidney disease in northeast of Iran: results of the Golestan cohort study. PLoS One.

[20] Ryan H, Trosclair A, Gfroerer J (2012). Adult current smoking: differences in definitions and prevalence estimates–NHIS and NSDUH, 2008. J Environ Public Health..

[21] Hemayatkhah M, Ghaffari S, Masjedi MR, Rahmanian V (2021). Frequency of tobacco use among students in Varamin city: results of the first phase of the PAD project study (tobacco use prevention in schools). Koomesh..

[22] Hemayatkhah M, Rahmanian V, Rahmanian K, Haghdoost A (2019). Population size estimation of groups at high risk of HIV/AIDS in men, using network scale up in south of Iran, 2017. J Isfahan Med Sch.

[23] Szklo M, Nieto FJ. Epidemiology: beyond the basics. USA: Jones & Bartlett Publishers; 2014.

[24] Graubard BI, Fears TR (2005). Standard errors for attributable risk for simple and complex sample designs. Biometrics..

[25] Safarinejad MR (2009). The epidemiology of adult chronic kidney disease in a population-based study in Iran: prevalence and associated risk factors. J Nephrol.

[26] Najafi I, Attari F, Islami F, Shakeri R, Malekzadeh F, Salahi R (2010). Renal function and risk factors of moderate to severe chronic kidney disease in Golestan Province, northeast of Iran. PLoS One.

[27] Najafi I, Shakeri R, Islami F, Malekzadeh F, Salahi R, Gharavi M (2012). Prevalence of chronic kidney disease and its associated risk factors: the first report from Iran using both micro albuminuria and urine sediment. Arch Iran Med.

[28] Naghibi M, Mojahedi MJ, Jarrahi L, Emadzadeh A, Ahmadi R, Emadzadeh M (2015). Prevalence of chronic kidney disease and its risk factors in Gonabad, Iran. Iran J Kidney Dis.

[29] Bakhshayeshkaram M, Roozbeh J, Heydari ST, Honarvar B, Dabbaghmanesh MH, Ghoreyshi M (2019). A population-based study on the prevalence and risk factors of chronic kidney disease in adult population of shiraz, Southern Iran. Galen Med J.

[30] Rao MK (2019). The utility of a National Surveillance Network to estimate CKD prevalence and identify high-risk populations in primary care. Kidney Int Rep.

[31] Tuttle KR, Alicic RZ, Duru OK, Jones CR, Daratha KB, Nicholas SB (2019). Clinical characteristics of and risk factors for chronic kidney disease among adults and children: an analysis of the CURE-CKD registry. JAMA Netw Open.

[32] Rowe JW, Andres R, Tobin JD (1976). Letter: age-adjusted standards for creatinine clearance. Ann Intern Med.

[33] Goncalves GMR, Silva EN (2018). Cost of chronic kidney disease attributable to diabetes from the perspective of the Brazilian unified health system. PLoS One.

[34] Poggio ED, Rule AD, Tanchanco R, Arrigain S, Butler RS, Srinivas T (2009). Demographic and clinical characteristics associated with glomerular filtration rates in living kidney donors. Kidney Int.

[35] Blake GM, Sibley-Allen C, Hilton R, Burnapp L, Moghul MR, Goldsmith D (2013). Glomerular filtration rate in prospective living kidney donors. Int Urol Nephrol.

[36] Chen K, Gosmanova E, Curhan G, Rejnmark L, Mu F, Swallow E (2019). MON-522 Risk of Chronic Kidney Disease (CKD) and Its Progression in Patients with Chronic Hypoparathyroidism (HypoPT). J Endocr Soc.

[37] Shen Y, Cai R, Sun J, Dong X, Huang R, Tian S (2017). Diabetes mellitus as a risk factor for incident chronic kidney disease and end-stage renal disease in women compared with men: a systematic review and meta-analysis. Endocrine..

[38] Khajehdehi P, Malekmakan L, Pakfetrat M, Roozbeh J, Sayadi M (2014). Prevalence of chronic kidney disease and its contributing risk factors in southern Iran a cross-sectional adult population-based study.

[39] Yu MK, Katon W, Young BA (2015). Associations between sex and incident chronic kidney disease in a prospective diabetic cohort. Nephrology..

[40] Wang J, Niratharakumar K, Gokhale K, Tahrani AA, Taverner T, Thomas GN (2022). Obesity without metabolic abnormality and incident CKD: a population-based British cohort study. Am J Kidney Dis.

[41] Ryu S, Chang Y, Woo H-Y, Kim S-G, Kim D-I, Kim WS (2008). Changes in body weight predict CKD in healthy men. J Am Soc Nephrol.

[42] Alarabi A, Nyström S-O, Ståhle E (1997). Acute renal failure and outcome of continuous arteriovenous hemodialysis (CAVHD) and continuous hemofiltration (CAVH) in elderly patients following cardiovascular surgery. Geriatr Nephrol Urol.

[43] Noshad S, Afarideh M, Heidari B, Mechanick JI, Esteghamati A (2015). Diabetes care in Iran: where we stand and where we are headed. Ann Global Health.

[44] Akbari F, Azadbakht L (2014). A systematic review on diet quality among Iranian youth: focusing on reports from Tehran and Isfahan. Arch Iran Med..

[45] Rezaei S, Mahmoudi Z, Sheidaei A, Aryan Z, Mahmoudi N, Gohari K (2018). Salt intake among Iranian population: the first national report on salt intake in Iran. J Hypertens.

[46] Bach KE, Kelly JT, Palmer SC, Khalesi S, Strippoli GF, Campbell KL (2019). Healthy dietary patterns and incidence of CKD: a meta-analysis of cohort studies. Clin J Am Soc Nephrol.

[47] Ku E, Lee BJ, Wei J, Weir MR (2019). Hypertension in CKD: core curriculum 2019. Am J Kidney Dis.

[48] Horowitz B, Miskulin D, Zager P (2015). Epidemiology of hypertension in CKD. Adv Chronic Kidney Dis.

[49] Lertpimonchai A, Rattanasiri S, Tamsailom S, Champaiboon C, Ingsathit A, Kitiyakara C (2019). Periodontitis as the risk factor of chronic kidney disease: mediation analysis. J Clin Periodontol.

[50] Kokubo Y, Nakamura S, Okamura T, Yoshimasa Y, Makino H, Watanabe M (2009). Relationship between blood pressure category and incidence of stroke and myocardial infarction in an urban Japanese population with and without chronic kidney disease: the Suita study. Stroke..

[51] Consortium CKDP (2010). Association of estimated glomerular filtration rate and albuminuria with all-cause and cardiovascular mortality in general population cohorts: a collaborative meta-analysis. Lancet.

[52] Van Der Velde M, Matsushita K, Coresh J, Astor BC, Woodward M, Levey AS (2011). Lower estimated glomerular filtration rate and higher albuminuria are associated with all-cause and cardiovascular mortality. A collaborative meta-analysis of high-risk population cohorts. Kidney Int.

[53] Said S, Hernandez GT (2014). The link between chronic kidney disease and cardiovascular disease. J Nephropathol.

[54] Jankowski J, Floege J, Fliser D, Böhm M, Marx N (2021). Cardiovascular disease in chronic kidney disease: pathophysiological insights and therapeutic options. Circulation..

[55] Podkowińska A, Formanowicz D (2020). Chronic kidney disease as oxidative stress-and inflammatory-mediated cardiovascular disease. Antioxidants..

[56] Vupputuri S, Soucie JM, McClellan W, Sandler DP (2004). History of kidney stones as a possible risk factor for chronic kidney disease. Ann Epidemiol.

[57] Kummer AE, Grams M, Lutsey P, Chen Y, Matsushita K, Köttgen A (2015). Nephrolithiasis as a risk factor for CKD: the atherosclerosis risk in communities study. Clin J Am Soc Nephrol.

[58] Shang W, Li L, Ren Y, Ge Q, Ku M, Ge S (2017). History of kidney stones and risk of chronic kidney disease: a meta-analysis. PeerJ..

[59] Yao KH, Guehi MC, Konan SD, Diopoh SP, Moudachirou MA, Sanogo S (2017). Prevalence and risk factors for chronic kidney disease in general population of Yopougon (Côte d’ivoire); a cross-sectional study. J Renal Inj Prev.

[60] Saber A, Tahami AN, Najafipour H, Azmandian J (2017). Assessment of prevalence of chronic kidney disease and its predisposing factors in Kerman city. Nephro-Urology Monthly..

[61] Wagner S, Merkling T, Metzger M, Bankir L, Laville M, Frimat L, et al. CKD-REIN study group. Water intake and progression of chronic kidney disease: the CKD-REIN cohort study. Nephrol Dial Transplant. 2022;37(4):730-9. 10.1093/ndt/gfab036 33576809

[62] Clark WF, Sontrop JM, Huang S-H, Gallo K, Moist L, House AA (2013). The chronic kidney disease water intake trial (WIT): results from the pilot randomised controlled trial. BMJ Open.

[63] Lebov JF, Valladares E, Peña R, Peña EM, Sanoff SL, Cisneros EC (2015). A population-based study of prevalence and risk factors of chronic kidney disease in León, Nicaragua. Can J Kidney Health Dis.

[64] Sontrop JM, Dixon SN, Garg AX, Buendia-Jimenez I, Dohein O, Huang S-HS (2013). Association between water intake, chronic kidney disease, and cardiovascular disease: a cross-sectional analysis of NHANES data. Am J Nephrol.

